# Singular Termination of Dropsy

**Published:** 1786

**Authors:** Robert Willan

**Affiliations:** Member of the Royal College of Physicians, and Physician to the Finsbury and Public Dispensaries in London


XIV. *Singular Termination of Dropſy. By* the ſame.

DEBORAH Hickman, aged thirty-eight years, a labouring woman, and the mother of ſeveral children, caught a ſevere cold about Chriſtmas laſt, which was ſoon ſucceeded by anaſarcous ſwellings of the legs. The catarrhal ſymptoms were relieved in a ſhort time by proper remedies, but the dropſical diſorder continued increaſing.

When I firſt ſaw her, March 30th, 1786, ſhe was univerſally bloated; her legs and thighs in particular were ſwelled to an enormous ſize: there ſeemed to be alſo an obſcure fluctuation, as from water effuſed in the abdominal cavity. On the 16th of April, two days after the periodical diſcharge had ceaſed, there came on a ſudden flow of water *per vaginam*, which drained through the bed before ſhe could get any aſſiſtance, and afterwards filled a veſſel, whoſe capacity was above three quarts, leaving her very faint and languid.

The evacuation continued in a more gradual manner for two days, when the cellular membrane ſeemed to be entirely unloaded. She ſoon after quitted her bed, and was able to follow her buſineſs as uſual. Within ten days the water was again accumulated in conſiderable quantity, when the ſame evacuation took place as before, and left her once more in a ſtate of inanition. She began now to take the bark, and went into the country.—The ſwellings have not ſince returned.

It is proper to obſerve, that ſhe took *pulv. digital. purpur.* gr. ij. three times a day for a fortnight prior to the firſt evacuation of water. This medicine had ſome effect in promoting the ſecretion of urine, but not conſiderably. The ſudden termination of the diſorder muſt be referred ferred to the operation of the remedy coinciding with the ftate of the uterine veffels, and determination of blood to that organ at the time.

In the following cafe, an effect nearly fimilar was produced by the *pulv. digitalis*, but through the natural excretory :

Mary Jenks, aged fifty-nine years, had been dropfical for five months. A cough and dyfpnœa came on in that time to fuch a degree, that fhe was unable to lie down in her bed. Her urine was in very fmall quantity. The *pulv. fcill. exfic.* was firft ordered, in dofes to be gradually increafed. After taking this for feveral weeks, no relief was obtained. She then took *pulv. digitalis purp.* as in the preceding cafe. On the fecond day it excited a prodigious flow of urine, which continued almoft inceffantly through that night and the fucceeding day. On vifiting her the next morning, I found the fwellings fubfided, and her difficulty of breathing removed. She thought the whole quantity evacuated could not be lefs than four gallons.

The fwellings have frequently returned fince that time; but, as foon as they become troublefome, fhe applies regularly for her medicine, which, taken in the dofe of gr. j. two or

 three three times a day, very ſoon removes the diſ-order.

*Hatton Street,*

May 10, 1786.

